# Central Melanocortins Regulate the Motivation for Sucrose Reward

**DOI:** 10.1371/journal.pone.0121768

**Published:** 2015-03-26

**Authors:** Rahul Pandit, Esther M. van der Zwaal, Mieneke C. M. Luijendijk, Maike A. D. Brans, Andrea J. van Rozen, Ralph J. A. Oude Ophuis, Louk J. M. J. Vanderschuren, Roger A. H. Adan, Susanne E. la Fleur

**Affiliations:** 1 Department of Translational Neuroscience, University Medical Center Utrecht, Utrecht, The Netherlands; 2 Department of Animals in Science and Society, Division of Behavioural Neuroscience, Faculty of Veterinary Medicine, Utrecht University, Utrecht, The Netherlands; 3 Department of Endocrinology and Metabolism, Academic Medical Center, University of Amsterdam, Amsterdam, The Netherlands; University of Leicester, UNITED KINGDOM

## Abstract

The role of the melanocortin (MC) system in feeding behavior is well established. Food intake is potently suppressed by central infusion of the MC 3/4 receptor agonist α-melanocyte stimulating hormone (α-MSH), whereas the MC 3/4 receptor inverse-agonist Agouti Related Peptide (AGRP) has the opposite effect. MC receptors are widely expressed in both hypothalamic and extra-hypothalamic brain regions, including nuclei involved in food reward and motivation, such as the nucleus accumbens (NAc) and the ventral tegmental area. This suggests that MCs modulate motivational aspects of food intake. To test this hypothesis, rats were injected intracerebroventricularly with α-MSH or AGRP and their motivation for sucrose was tested under a progressive ratio schedule of reinforcement. Food motivated behavior was dose-dependently decreased by α-MSH. Conversely, AGRP increased responding for sucrose, an effect that was blocked by pretreatment with the dopamine receptor antagonist α-flupenthixol. In contrast to progressive ratio responding, free intake of sucrose remained unaltered upon α-MSH or AGRP infusion. In addition, we investigated whether the effects of α-MSH and AGRP on food motivation were mediated by the NAc shell. In situ hybridization of MC3 and MC4 receptor expression confirmed that the MC4 receptor was expressed throughout the NAc, and injection of α-MSH and AGRP into the NAc shell caused a decrease and an increase in motivation for sucrose, respectively. These data show that the motivation for palatable food is modulated by MC4 receptors in the NAc shell, and demonstrate cross-talk between the MC and dopamine system in the modulation of food motivation.

## Introduction

The central melanocortin (MC) system, which functions downstream of the leptin pathway, forms an integral part of the hypothalamic feeding circuitry [1]. Within the arcuate nucleus of the hypothalamus, pro-opio melanocortin (POMC) neurons express α-melanocyte stimulating hormone (α-MSH), an agonist for the MC3 and MC4 receptors. A neighboring neuronal population expresses Agouti Related Peptide (AGRP), an inverse agonist for the same MC receptors [1–4]. The opposing effects of α-MSH and AGRP on feeding are well established.

In states of hunger, hypothalamic α-MSH levels are decreased while AGRP levels are increased [5,6], whereas the reverse pattern is observed in states of obesity or positive energy balance following overfeeding [7;8]. In line with these observations, central infusion of α-MSH or its cyclic analogue MTII robustly suppresses food intake [9;10], whereas treatment with AGRP or the MC3/4 receptor antagonist SHU9119 has the opposite effect [9;11]. These studies clearly demonstrate the role of MCs in food intake. However, it remains incompletely understood to what extent these effects are the result of changes in hunger, satiety or motivation to obtain food.

Animals exposed to an obesogenic diet display increased motivation for food accompanied by lower POMC levels [12;13]. Furthermore, MC4 receptor knock-out mice become obese and exhibit augmented food-motivated behavior in non-food-deprived states [14]. Together, these findings suggest that lower MC receptor activity is associated with an enhanced motivational drive for food. Thus, there is some evidence that MC ligands modulate the motivation for food [15;16], but the neural mechanisms underlying these effects remain unclear.

One brain area of particular interest is the nucleus accumbens (NAc), through which dopaminergic neurotransmission regulates food-motivated behavior [17]. Activation of the NAc shell has been reported in animals lever pressing for sucrose pellets under a progressive ratio schedule of reinforcement [18]. Moreover, central infusion of AGRP leads to activation of the NAc shell, indicating that the behavioral effects of AGRP involve this brain area [19]. Intriguingly, overexpression of AGRP in the NAc shell has no effects on food-intake and body weight [20], suggesting that the NAc mediates other effects of MCs on eating, such as motivation for food and food palatability. Combined, these pieces of evidence identify the NAc shell as a candidate region to mediate the effects of MCs on motivation for food.

Importantly, food reward or reward-related stimuli have been shown to increase dopamine levels within the NAc, whereas responding for a palatable food reward was inhibited by administration of a dopamine receptor antagonist into the NAc [21;22]. We therefore hypothesized that the effects of MC ligands on food-motivated behavior are dopamine mediated. Support for this hypothesis is provided by studies showing that the NAc receives projections from hypothalamic MC neurons and abundantly expresses MC4 receptors [2;23–26]. In addition, microdialysis experiments have shown that, compared to the NAc core, the NAc shell is more responsive to dopamine increases when animals are engaged in an operant task [27].

In the present paper, we therefore investigated the role of the MC system in food motivation by determining the effect of central infusion of MC 3/4 receptor ligands on the motivation to obtain a palatable food reward (i.e., sucrose) under a progressive ratio schedule of reinforcement [28;29]. The involvement of dopamine neurotransmission in the effects of MC 3/4 ligands on food-motivation was determined using the dopamine receptor antagonist α-flupenthixol. Finally, in order to determine whether the NAc shell mediates the effect of MC signaling on food reward, we first assessed whether MC receptors were expressed within the NAc shell and subsequently studied the behavioral effects of MC ligands injected directly into the NAc shell.

## Materials and Methods

### Animals

Male Wistar rats (Charles River, Sulzfeld, Germany) weighing 200–225 g on arrival were individually housed (Macrolon cages; 40x26x20 cm) with *ad libitum* access to rat chow (3.31Kcal/gram, Standard Diet Service, UK) and tap water. Animals were kept in a temperature (21±2°C) and humidity (60–70%) controlled room under a 12 h reversed light/dark cycle (lights on at 19.00 h). All experimental procedures were approved by the Animal Ethics Committee of Utrecht University and were in agreement with Dutch laws (Wet op Dierproeven 1996) and European regulations (Guideline 86/609/EEC).

### Surgery

Surgery was performed when animals weighed between 275-300g. Rats were anaesthetized with 0.1 ml/100 g i.m. fentanyl/fluanisone (Hypnorm, Janssen Pharmaceutica, Belgium). For implantation of intracerebroventricular (i.c.v.) cannulas, the head was shaved and the skull exposed by a midline incision of the skin. After preparation of a small craniotomy (approximately 1 mm in diameter), a 5 mm stainless steel guide cannula (Plastics One, USA) was inserted into the lateral ventricle (1 mm lateral and 1 mm posterior from bregma). Cannulas were fixed to the skull with two stainless steel screws and dental cement. For implantation of cannulas aimed at the NAc shell, animals were positioned in a stereotaxic apparatus (David Kopf, USA) and stainless steel guide cannulas (26 GA, 8 mm; Plastics One, USA) were implanted bilaterally, 1 mm above the NAc shell. Coordinates relative to bregma were as follows (in mm): AP: +1.20 ML: + 2.80 DV:- 7.50, angle 10° (Paxinos and Watson, 1998). Perioperatively, rats received carprofen (5 mg/kg s.c.) and saline (3 ml s.c.). Behavioral experiments commenced following a 10–14 days recovery period.

At the end of the experiment, cannula placement was verified using histological methods. Animals equipped with i.c.v. cannulas were euthanasized with carbon dioxide and received an i.c.v. injection with 2 μl of ink. Brains were then removed and the lateral ventricles opened to check for ink staining. Rats equipped with NAc shell cannulas were decapitated and their brains removed and frozen on dry ice. Next, cryostat sections (16 μm) were stained with cresyl violet to determine cannula locations. Data from animals with cannula placements outside the target area were excluded from the analysis.

### Drugs and microinfusions

α-MSH (Bachem GmbH, Germany) and AGRP (83–132) Amide (Phoenix Pharmaceuticals, USA) were dissolved in sterile saline. For i.c.v. infusions, rats were briefly restrained and 2μl of drug solution was slowly (10–15 seconds) injected into the ventricular cavity. Infusions into the NAc shell were performed through an injector (8 mm, 33 GA, Plastics One, USA) inserted into the guide cannula. Bilateral infusions (300 nl over 30 sec) were given using a syringe pump with the injectors left in place for another 30 sec to allow for diffusion. Behavioral testing commenced 5 min after drug infusions. α-flupenthixol dihydrochloride (Sigma Aldrich, USA) was dissolved in saline and injected i.p. 30 min prior to i.c.v. infusions. All animals received all drug doses/combinations, according to a latin square design. Furthermore, a minimum 2-day drug-free period was maintained between infusions during which the animals were trained but not tested.

### Experimental design

#### Central effects of α-MSH and AGRP on motivation for sucrose

**Apparatus**: Operant conditioning chambers (30.5 cm x 24.1cm x 21.0 cm; Med-Associates, USA) situated in light- and sound-attenuating cubicles equipped with a ventilation fan were used. Each chamber had a metal grid floor, two retractable levers with white stimulus lights above it and a food dispenser, which delivered 45 mg sucrose pellets (Noyes Precision Pellets Formula F, Research Diets, USA) to the food receptacle. Chambers were illuminated by a white house light. Data collection and processing was controlled by MED-PC software.

**Training**: Following recovery from surgery, the rats were first trained under a fixed ratio (FR) 1 schedule of reinforcement, with 2 sessions/day for five consecutive days. Under this schedule, a single press on the active lever resulted in the delivery of one 45 mg sucrose pellet, illumination of the light above the lever and retraction of the lever. Twenty seconds after the pellet was received, the lever was reinserted into the chamber. Sessions lasted 30 min or until rats earned 60 pellets, whichever occurred first. Presses on the inactive lever were recorded, but had no programmed consequences. Positions of the active and inactive levers were counterbalanced between animals. After 10 sessions under the FR1 schedule, the progressive ratio (PR) schedule of reinforcement was introduced. Under a PR-schedule, the cost of a reward is progressively increased over successive trials to determine the effort the rat will emit for it. The response requirement increased according to the following equation [28;29]: response ratio = (5 X e^(0,2 x infusion number)^)– 5 through the following series: 1, 2, 4, 6, 9, 12, 15, 20, 25, 32, 40, 50, 62, 77, 95, 118, 145, 178, 219, 268, 328, 402, 492, 603, 737. The session ended when the animal failed to earn a reward for 60 min. Responding was considered stable when the number of food pellets earned per session did not differ more than 15% for three consecutive sessions and up- or downward trends were absent. After 10 training sessions, rats were tested during a period of 3–5 weeks with 5–6 training sessions/week including training on non-testing days. All behavioral testing was performed between 8.00 and 17.00 h.

In a group of 12 rats, the effects of α-MSH on PR-performance were tested. On test days, rats received either saline (2 μl) or α-MSH (0.66 nmol or 1.2 nmol) according to a latin square design. Data from 5 rats were excluded from analysis due to instable responding under the PR schedule (3 rats) and misplaced cannulas (2 rats).

To study the effects of AGRP on responding for sucrose under the PR schedule of reinforcement, a separate group of 12 rats were either infused with saline or AGRP (0.66 nmol or 1.0 nmol) according to a latin square design. Data from 5 rats were excluded from analysis due to instable responding under the PR schedule (2 rats), unexplained weight loss following testing (2 rats) and a misplaced cannula (1 rat).

#### Central effects of α-MSH and AGRP on free-feeding of sucrose

Free-feeding experiments were conducted to determine whether the effects of α-MSH and AGRP were specific for the animal’s motivation for palatable food or secondary to an effect on feeding behavior in general. In this setup, animals had free access to sucrose pellets for 60 min every day, i.e. the animals did not need to perform an operant task to obtain the sucrose pellets. Ad-libitum fed rats were introduced into an empty cage with a suspended steel receptacle containing 45 mg sucrose pellets. Sessions took place in the beginning of the dark period. After 1 h, sucrose pellet intake was measured. To accustom rats to the procedure and stabilize sucrose intake, 4–5 training sessions were performed before testing.

A separate group of 12 rats was used to determine the effects of α-MSH on free-feeding of sucrose. Rats were infused with saline (2 μl) and two doses of α-MSH (0.66 nmol or 1.2 nmol) according to a latin square design. Data of 5 rats were excluded from analysis due to unexplained weight loss after injection and testing (4 rats) and a misplaced cannula (1 rat).

In a separate experiment, effects of AGRP on free-feeding of sucrose were studied. Twelve rats were infused with saline (2 μl) and AGRP (0.66 nmol or 1 nmol) according to a latin square design. Data of 3 animals were excluded from analysis due to unexplained weight loss following i.c.v. injection.

#### Interaction between dopamine and MC systems in the motivation for sucrose

To investigate the role of dopamine in the effects of AGRP on food-motivated behavior, a pilot experiment was first performed to determine the dose of the dopamine receptor antagonist α-flupenthixol to be used. A separate group of rats (n = 6) was injected with α-flupenthixol according to a latin square design (all rats received 0, 0.125, 0.25, 0.5 mg/kg i.p.) 30 minutes prior to a PR session (data not shown). The dose used for the interaction experiment (0.125 mg/kg) was chosen because it had no effect on PR performance, whereas higher doses reduced responding under a PR-schedule of reinforcement [30].

A separate group of eighteen rats was injected i.p. with saline or α-flupentixol (0.125 mg/kg, 0.5 ml) 30 min prior to i.c.v. saline or 1 nmol of AGRP infusion according to a latin square design. Five minutes after i.c.v. infusions, responding for sucrose under a PR schedule was tested as outlined above. Data of 6 rats were excluded from analysis due to unexplained weight loss after injection (1), misplaced cannulas (1), and unstable responding during training and testing (4).

### The role of the NAc in MC-dependent motivation for sucrose

#### Fluorescent *in-situ* hybridization to characterize MC4 receptor expression in the NAc

In order to understand whether the effect of α-MSH and AGRP on food motivation is mediated through the NAc, we first studied the expression patterns of MC3 or MC4 receptors in the NAc, and whether they were expressed by dopamine D1 and/or D2 receptor positive cells. For this purpose, a triple *in-situ* hybridization experiment with 3 different fluorophores was performed.

For this experiment, a naive group of rats was used that was not subjected to behavioral testing. Following decapitation, brains were frozen on dry ice. Coronal sections (16 μm) were fixed with 4% paraformaldehyde (10 min), subsequently washed with PBS (3x, 10min) and further acetylated (10 min). Following acetylation, slices were preincubated with a prehybridization mix (50% deionized formamide, 5x SSC, 5x Denhardt's solution, 250 μg/ml tRNA baker's yeast, 500 μg/ml sonicated salmon sperm DNA final concentrations in MilliQ). Next, sections were incubated overnight at 40°C in 120 μl hybridization mix (Panomics) containing the probe sets (concentration D1/D2 1:33, MC3 receptor/MC4 receptor 1:50) and washed. Probe sets used to detect the desired rat mRNAs we designed by Panomics (Santa Clara, USA), using published sequences (see S1 table and [31]). The probes were designed to hybridize at adjacent regions of the target mRNA, allowing the hybridization of a preamplification oligo (Panomics) that spans the hybridized probe pair, thus ensuring signal specificity. This specific signal was further amplified by hybridization of amplification oligo's (Panomics) and visualized by label oligo's (Panomics), resulting in an amplification of up to 500 times for low abundant mRNAs.

Sections were then incubated for 1.5 h at 40°C in 120 μl PreAmplifier Mix (Panomics) containing preamplification oligos (PreAmp TYPE4 1:20, PreAmp TYPE 6 1:50, PreAmp TYPE 8 1:33) and washed. Then, sections were incubated for 1.5 h at 40°C in 120 μl Amplifier Mix (Panomics) containing amplification oligos (Amp TYPE4 1:20, Amp TYPE 6 1:50, Amp TYPE 8 1:33) and washed. Next, sections were incubated for 1.5 h at 40°C in in 120 μl in Label Probe Mix (Panomics) containing label oligos (LP TYPE4 1:20, LP TYPE 8 1:33, LP TYPE 6 1:50). After washing, sections were incubated in 750 μl PBS supplemented with 4',6-diamidino-2-phenylindole (DAPI) (6.7 μg/ml, Sigma Aldrich, St. Louis, USA) for 5 min. Finally, slices were washed and embedded in Mowiol. Sections were visualized and images were obtained with a Zeiss AxioScope A1 microscope (Carl Zeiss, Jena, Germany) equipped with Chroma filter sets (Chroma, Bellows Falls, USA) and Zeiss AxioVision Rel. 4.8 acquisition software. DAPI images were acquired using the 31000v2 filter block of Chroma. The Chroma FITC filter block 410001 was used to acquire the 488 nm conjugated TYPE4 label. The 550 nm conjugated TYPE8 label was acquired using a Chroma TRITC 41002b filter block containing a narrowband excitation filter. A custom Chroma Cy5 infrared filter was used for the acquisition of the 650 nm conjugated TYPE6 label. This label was excited at 650 nm using an HQ650/45x filter (Chroma) and light was directed by a Q680LP dichroic mirror (Chroma) through a HQ690LP emission filter (Chroma). Images were processed and analyzed using ImageJ 1.43r software.

The (co)expression of the mRNA transcripts of the different receptors was quantified within the core and shell sub-regions of the NAc. At least 8 images per sub-region, spanning the entire rostro-caudal axis of the NAc (300 μm x 220 μm) were used for quantification of mRNA with the *DRD1* and *DRD2* transcripts. Cells were identified on the basis of nuclear DAPI staining and were counted as expressing a certain mRNA if one or more fluorescent dots were present in, or in close vicinity of (defined as a circle with twice the diameter of the DAPI staining) the area of DAPI staining. The percentage of cells expressing a certain mRNA was determined by dividing the amount of cells expressing a certain mRNA by the total amount of DAPI stained nuclei. The percentage of cells co-expressing dopamine D1 or the dopamine D2 receptor mRNA with MC3 receptor or MC4 receptor mRNA was determined by dividing the amount of cells positive for the dopamine D1 or the dopamine D2 receptor and MC3 receptor/MC4 receptor mRNA by the total amount of dopamine D1 and dopamine D2 receptor positive cells.

#### POMC fibers innervate the NAc Shell

In order to study whether POMC neurons innervate the NAc, we performed an immunohistochemistry study. Two rats were perfused with ice-cold 0.9% NaCl followed by 4%PFA in PBS. Following perfusion, brains were removed, post-fixated overnight in 4%PFA, and then embedded in 30% sucrose solution in PBS. Free-floating 40μm slices were stained for POMC peptide. Briefly, slices were washed with 0.5M Tris-Buffered solution (TBS) (3x10min), and then blocked with super mix (0.5M TBS, 0.25% gelatin and 0.1%TritonX) for 30 minutes. Following blocking, slices were incubated with primary antibody against POMC (Rabbit, H-029-30, Phoenix Pharmapseuticals, USA) (4°C, overnight) on a shaker. Subsequently slices were washed (TBS, 3x10min) and incubated with fluorescent secondary antibody (chicken anti rabbit, 488, A-21441 Alexa Fluor, Life Technologies, USA) for 1 hour at room temperature. Slices were further washed (3x10min) with TBS, put on glass slides and embedded with Flurosave (Merk Milipore, USA). Images were made using Zeiss AxioScope A1 microscope (Carl Zeiss, Jena, Germany) and processed with ImageJ sofatware.

#### Effects of NAc infusions of α-MSH and AGRP on motivation and free-feeding of sucrose

To determine the role of the NAc shell in the effects of α-MSH and AGRP on the motivation for sucrose, animals received bilateral infusions of saline and α-MSH (0.2 nmol) or AGRP (0.1nmol) in a counterbalanced fashion (300nl in 30 seconds). Subsequently, the animals were tested as outlined in earlier. Data from 1 animal was excluded from the final analysis due to incorrect cannula placement.

Free-feeding experiments were conducted in a separate group of rats as described in earlier. Here, 11 animals received bilateral NAc shell infusions with saline, α-MSH (0.2 nmol) and AGRP (0.1nmol) in a counterbalanced manner and tested for sucrose consumption for one hour.

### Statistical analyses

For the final analyses, data were excluded from animals with 1) instable responding under the PR schedule of reinforcement (i.e., > 15% variation between three consecutive sessions and/or a consistent upwards or downwards trend in the number of rewards earned); 2) unexplained body weight loss (>20 grams); 3) incorrect cannula placements. Unless otherwise indicated, data from all experiments were analyzed using repeated measures ANOVA followed by a Tukey *post-hoc* test where appropriate. Data from experiment with α-flupentixol were analyzed using two-way repeated measures ANOVA with i.p. injections (saline vs α-flupentixol) and i.c.v. infusions (saline vs AGRP) as within subject’s factors. Data from experiments with infusions of MC ligands in the NAc shell were analyzed using a paired samples t-test. Differences were considered significant at p<0.05. All statistical analyses and graphical representations were performed using Graphpad software (v 6.03, USA). All data are expressed as Mean ± SEM.

## Results

### Central effects of α-MSH and AGRP on the motivation for sucrose

**Fig 1:**
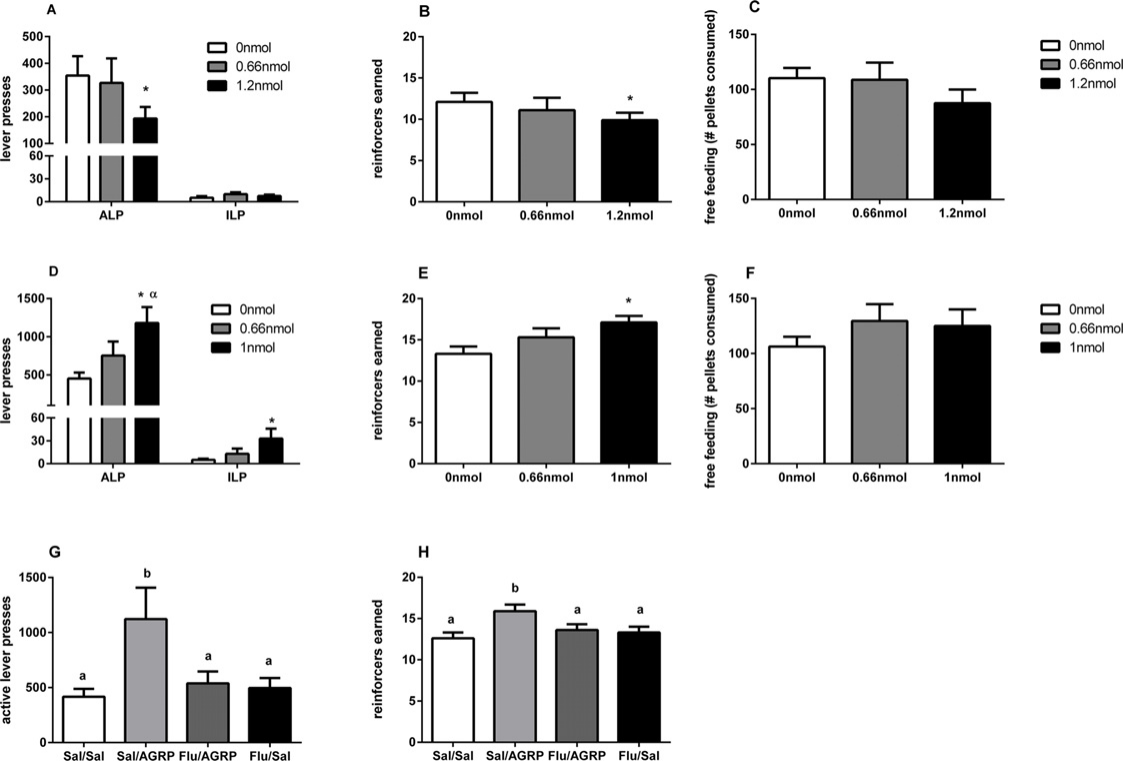
Effect of α-MSH and AGRP on motivation and sucrose free feeding. Lever presses, rewards earned and number of sucrose pellets consumed under free feeding conditions upon central infusion of α-MSH (**A-C**) or AGRP (**D-F**). Active lever presses (**G**) and rewards earned (**H**) during a PR schedule of reinforcement upon pretreatment with systemic saline or α-flupenthixol (0.125mg/kg) prior to central AGRP (1ηmol/2μl) or saline (2μl) infusion. Data are mean ± SEM. * denotes statistically significant difference (p<0.05) between saline and test conditions. α denotes statistically significant difference between the two test conditions.

I.c.v. infusion of α-MSH (1.2 nmol) significantly reduced both the number of active lever presses (F_(2,12)_ = 4.5, p<0.05, Fig. 1A) and rewards earned (F_(2,12)_ = 6.0, p<0.05, Fig. 1B). The amount of inactive lever presses following infusion of α-MSH remained unchanged (F_(2,12)_ = 5.0, p>0.05, Fig. 1A). Conversely, the highest dose of AGRP (1.0 nmol) significantly increased the number of active lever presses (F_(2,12)_ = 10.6, p<0.05, Fig. 1D) and rewards earned (F_(2,12)_ = 11.5, p<0.05, Fig. 1E). The number of inactive lever presses following infusion of AGRP was enhanced (F_(2,12)_ = 5.0, p<0.05, Fig. 1D).

### Central effects of MCs on free-feeding of sucrose

In the free-feeding paradigm, infusion of α-MSH or AGRP had no effects on sucrose intake (F_(2,24)_ = 2.3, p>0.05, Fig. 1C), (F_(2,28)_ = 3.1, p>0.05, Fig. 1F).

### Interaction between dopamine and MC systems in the motivation for sucrose

In order to assess the interaction between the MC and dopaminergic systems, rats were pre-treated with either α-flupenthixol or saline, prior to infusion of saline or 1nmol of AGRP. Infusion of 1nmol of AGRP increased the number of active lever presses and rewards earned under the PR schedule of reinforcement. The effect of AGRP on both these measures was attenuated by pre-treatment with α-flupenthixol, whereas α-flupenthixol failed to affect PR performance on its own (Fig. 1G and Fig. 1H), Two-way repeated measures ANOVA revealed a significant effect of AGRP on both active lever presses (F_(1,11)_ = 8.4, p<0.05) and rewards earned (F_(1,11)_ = 18.2, p<0.05). There was a significant interaction between α-flupentixol treatment and AGRP infusion for the number of rewards earned (F_(1,11)_ = 9.4, p<0.05), and a trend towards an interaction for the number of active lever presses (F_(1,11)_ = 3.4, p = 0.08).

### Expression of MC4 receptors but not MC3 receptors in the NAc

**Fig 2:**
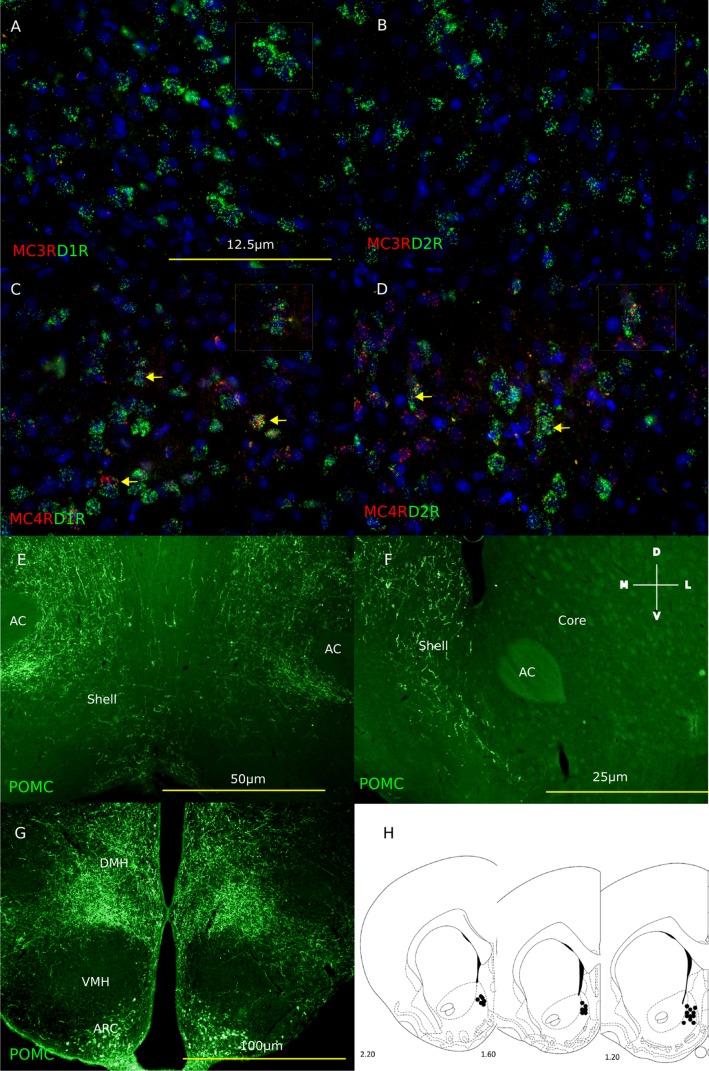
Melanocortin receptor signaling in the nucleus accumbens. Fluorescent *in-situ* hybridization with MC3 and MC4 receptors on rat brain slices containing the NAc shell. Nuclei were stained with DAPI and pseudocolored in blue and MC receptors are shown in red and D1/D2 receptors in green. Arrow indicates cells with prominent mRNA co-localization (**A-D**). POMC expression in the ARC (Bregma -3.30mm) (**G**) and in the NAc shell but not core regions (Bregma = +1.2mm) (**E and F**). Cannula placements within the NAc shell. Numbers indicate relative location from the bregma (**H**).

To understand whether MC ligands exert their effects on food motivation via the NAc, fluorescent in-situ hybridization was conducted for the MC3 receptor and MC4 receptors. In line with literature, Fig. 2A-D shows prominent expression of MC4 but not MC3 receptors in the NAc [32]. Co-localization of MC4 receptors was observed with both dopamine D1 and D2 receptors. The distribution of MC4 receptor expressing cells was uneven and showed a decreasing gradient from ventral to dorsal parts of the striatum. The percentage of MC4 receptor positive cells in the NAc shell and core was 5.7 ± 1.6% and 8.3 ± 1.8% respectively. Within the NAc shell, 12.9 ± 3.3% of MC4 receptors were co-expressed in dopamine D1 expressing neurons and 10.9 ± 2.4% within dopamine D2 receptor positive neurons. These data support a postsynaptic interaction between MC4 receptor and dopamine D1/D2 receptors, although a presynaptic interaction cannot be entirely excluded. Furthermore, Fig. 2E-G identifies POMC neuronal projections specifically in the shell region of the NAc, indicating the NAc shell as a crucial site for MC action.

### The role of the NAc shell in MC-dependent motivation for sucrose

**Fig 3:**
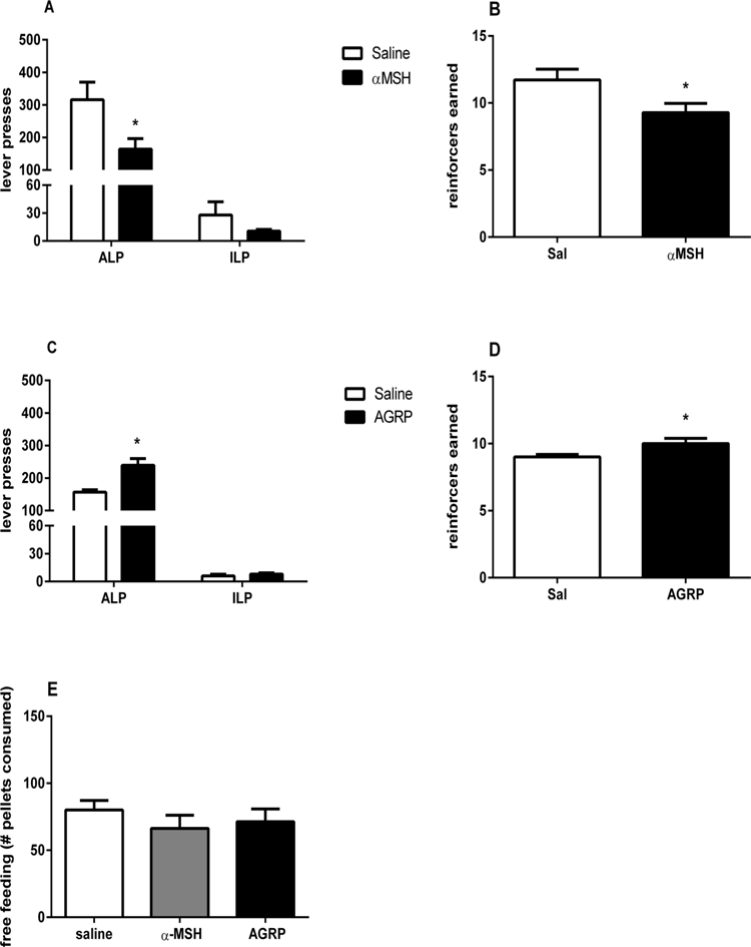
Effects of intra-NAc α-MSH and AGRP infusion on motivation and free-intake. Lever presses and rewards earned during a PR schedule of reinforcement uponintra NAc injections of α-MSH (**A and B**) or AGRP (**C and D**). Number of 45 mg sucrose pellets consumed under free-feeding conditions following central infusion of saline, α-MSH and AGRP (**E**). Data are mean ± SEM. * denotes statistically significant difference (p<0.05) between saline and test conditions.

Bilateral infusion of 0.2 nmol of α-MSH into the NAc shell decreased both the number of active lever presses (t(6) = 2.9, p<0.05) and reinforcers earned (t(6) = 2.7, p<0.05) without affecting the number of inactive lever presses (p>0.05, Fig. 3A and Fig. 3B). Conversely, infusion of 0.1nmol of AGRP increased both active lever presses (t(7) = 4.1, p<0.05) and reinforcers obtained (t(8) = 3.5, p<0.05, Fig. 3C and Fig. 3D) without affecting the number of inactive lever presses (p>0.05). Free-feeding of sucrose was not significantly affected by 0.2 nmol of α-MSH or 0.1nmol of AGRP (F_(2,20)_ = 0.9, p>0.05, Fig. 3E). Placement of the NAc canulas is depicted in Fig. 2H.

## Discussion

Here we show that rats treated i.c.v. with α-MSH show decreased motivation for sugar, while infusion of AGRP had the opposite effect. Furthermore, increase in responding for sucrose by AGRP was blocked by α-flupenthixol, indicating that this stable effect of AGRP was dopamine dependent. We further identify the NAc shell as an important site of action for the motivational effects of MCs. Thus, the NAc shell is innervated by POMC-positive fibers and expresses MC4, but not MC3 receptors. In addition, infusion of α-MSH and AGRP into the NAc shell mimics their i.c.v. effects on responding for sucrose, indicating that MC signaling in the NAc shell modulates the motivation for palatable food.

The willingness to work for a food reward is thought to be one of the mechanisms through which MCs influence feeding [15;16;20]. Here, we therefore investigated the role of the MC system on the motivation for sucrose in *ad libitum* fed rats.

As mentioned previously, food restriction results in alterations in neuropeptidergic signaling involved in feeding [33] and as a result, alterations in food-motivated behavior in general [34]. We therefore chose to infuse MC ligands under *ad libitum* conditions. In this manner, we aimed to avoid any confounding effects of food restriction on food motivation and to specifically determine the effect of MC receptor activation on the motivation for palatable food.

Our findings confirm that alterations in food-motivated behavior play a role in the effects of the MC system on the regulation of food intake. Rats that received i.c.v. infusions with MC ligands demonstrated opposing effects on motivation, with α-MSH suppressing operant responding for sucrose and AGRP increasing it. These results are in line with previous findings from other laboratories, where, in addition to enhancing chow intake, central AGRP infusion has also been shown to increase motivation for both sugar [16] and fat [35].

In order to determine whether central MCs influenced sugar intake in general as well as food motivation, free feeding experiments were also conducted. In these experiments, animals could freely consume sucrose pellets without having to perform an operant task. Our results failed to demonstrate any effects of α-MSH or AGRP on free feeding of sucrose, indicating that MCs can modulate motivation independent of food intake. The lack of effect of AGRP might seem surprising as AGRP is known to stimulate (palatable) feeding. However, most evidence is based on studies using fat feeding [35]. It could therefore well be that sugar intake is differentially modulated by MC signaling. Indeed, in rats given the choice between saturated fat, liquid sugar and chow, AGRP enhances chow and saturated fat intake but not sugar intake (unpublished data, see S1 Fig.). The finding that sucrose intake is influenced by AGRP under a PR schedule of reinforcement but not under free feeding conditions, further supports the notion that AGRP influences food-motivation in general. Nevertheless based on the current experimental paradigm, possible α-MSH effects on sucrose intake at a different time point cannot be excluded.

In order to identify the neural substrates underlying MC-driven food motivation, we focused on the NAc shell, as its role in mediating motivation for rewards is well known [36]. Our results show that although MC signaling within the NAc shell does not affect chow intake and body weight [20], it does regulate motivation for palatable food. It is known that the NAc receives projections from dopaminergic neurons in the VTA and from hypothalamic POMC neurons [2;23–26;32;37]. Furthermore, the NAc shell but not the core region is activated in rats responding for food under a PR schedule of reinforcement [18]. Similarly, central infusion of AGRP increases activity of the shell region of the NAc [19]. In line with these findings, our immunohistochemistry study shows extensive POMC neuronal fibers in the shell region only, indicating the importance MC signaling within the shell region. Using fluorescent *in situ* hybridization techniques, we first demonstrated pronounced expression of MC4 but not MC3 receptors in this region. In addition, MC4 receptors were expressed on both dopamine D1 and D2 receptor expressing neurons. Interestingly, activation of D1-positive neurons within the NAc is associated with an enhanced behavioral response to drug reinforcers [38]. Conversely, optogenetic inhibition of these neurons or infusion of a D1 receptor antagonist into the NAc decreases responding for sucrose and cocaine [39;40].

The role of dopamine in the motivation for food reward is well established [21;36;41]. For instance, ingestion of sucrose increases dopamine levels within the NAc [42], whereas blocking dopamine signaling decreases operant responding for sucrose, especially when response requirements are high [17]. Since AGRP infusion enhanced operant responding for sucrose, we hypothesized that the effect of AGRP on the motivation for sucrose was mediated by dopamine and thus would be blocked by pretreatment with a dopamine receptor antagonist. Indeed, the AGRP-induced increase in operant responding for sucrose under a PR schedule was prevented by a low dose of α-flupenthixol that failed to affect general locomotion or motivation for sucrose on its own. These data suggest that AGRP-enhanced motivation for palatable food rewards is dopamine dependent, which is in line with the finding that infusion of MC agonists modulates dopamine receptor levels in various brain areas [43].

Although not studied extensively, MC signaling in the NAc has been associated with attenuation of excitatory postsynaptic currents and synaptic strength. Lim et al. reported a decrease in AMPA/NMDA ratio specifically in dopamine D1 but not D2 receptor expressing NAc neurons following α-MSH treatment, indicating decreased activity of these neurons after α-MSH treatment [44]. Based on these findings [39;44] and our current observations, it is reasonable to speculate that α-MSH acts on the dopamine D1 receptor-positive population of neurons within the NAc to attenuate motivation for sucrose, and that antagonizing MC4 receptors with AGRP results in opposite behavioral effects. However, our data also demonstrate MC4 and dopamine D2 receptor co-localization, and α-flupenthixol acts on both D1 and D2 receptors. Thus, it is possible that melanocortin signaling in D2 positive neurons partly mediate melanocortin dependent motivation. Taken together, these data suggest that the NAc mediates the effect of MCs on food-motivated behavior. Consistent with our hypothesis, intra-NAc infusion of α-MSH and AGRP mirrored their effects on motivation for sucrose after i.c.v infusion, indicating that MC action within the NAc is sufficient to modulate food-motivated behavior.

Interestingly, in the free-feeding paradigm of the present study, infusion of MC ligands into the NAc failed to alter the consumption of sucrose. This is in line with the finding that overexpression of AGRP [20] and infusion of a MC4 antagonist [39] within the NAc does not alter food-intake or meal patterns.

In summary, our findings further identify the NAc shell as a prime target for MC action and demonstrate an interaction between dopamine and MCs in the motivation for palatable food. Our data provide a clear picture of the motivational effects of MCs on food reward, thereby adding to the existing knowledge of their role in food satiation [20;45], anticipation [46] and consumption [9].

## Supporting Information

S1 Dataset Final data used for statistical analysis. (XLSX)

S1 Fig Effect of AGRP injected in the lateral ventricle on 5h chow, fat and sugar intake in rats on a fcHFHS diet. Fat intake was significantly increased, whereas chow and sugar intake were not affected by AGRP. Rats were injected in a cross over design, and order of injection did not affect outcome. (Data are mean ± SEM and *: p<0.05 with paired t-test).
(DOCX)

S1 Table Regions and accession numbers used for synthesis of the FISH probes. (DOCX)
