# Supplementary material for: Central Melanocortins Regulate the Motivation for Sucrose Reward
Source: PLoS One. 2015 Mar 26;10(3):e0121768. doi: 10.1371/journal.pone.0121768 (PMC4374756; doi:10.1371/journal.pone.0121768)
Supplement: S1 Fig — Fat intake was significantly increased, whereas chow and sugar intake were not affected by AGRP. Rats were injected in a cross over design, and order of injection did not affect outcome. (Data are mean ± SEM and *: p<0.05 with paired t-test). (DOCX) [file pone.0121768.s002.docx]

*S2 Figure:* **Effect of AGRP infusion in the lateral ventricle on food intake when rats are subjected to a free-choice high-fat high-sugar diet.** Fat intake was significantly increased, whereas chow and sugar intake were not affected by AGRP. Rats were injected in a cross over design, and order of injection did not affect outcome. (Data are mean ± SEM and *: p<0.05 with paired t-test).

**Method:** After recovery from ICV surgery (as described in manuscript), rats were switched to a free choice high-fat high sugar diet (fcHFHS) for which rats were exposed to a cup of saturated fat (ossewit, vandermorteelte, belgium), a bottle of 30% sucrose water, a bottle of tap water and familiar chow (see for description of this diet: la Fleur et al, Int J Obes 2010&2011). After a week on the diet, rats were injected with 2ul saline icv to get accustomed to the method.

Seven days later, at the beginning of the light peroid, 4 rats were injected with saline and 5 rats with AGRP (83-132) Amide (5.6ug/2ul saline) (Phoenix Pharmaceuticals Inc.,Burlingame, USA) and 5h and 24h later lard, sugar and chow intake were measured. Four days later, a similar experiment was performed, this time injecting the saline animals with AGRP and vice versa. In this way every animal was used as its own control. At the end of the experiments, rats were decapitated and brains were checked for canula placement. Data from 2 of the 9 animals were not included in the analysis due to unexplainable weight loss after the icv infusion and a misplaced canula.

**Results:** AGRP (1nmol) injected in the lateral ventricle increased 5h caloric intake in rats on the fcHFHS diet significantly (20.0 kcal ± 2.7 in saline injected rats compared to 35.4 kcal ± 4.3 in AGRP injected rats; p<0.04). Below the effects of AGRP on chow, fat and sugar are depicted at the 5h time point. AGRP significantly increased fat intake compared to a saline injection, whereas chow and sucrose intake were not affected by AGRP (Fig 1A). After 24h, fat intake was still significantly increased in AGRP injected rats and intake of chow and sucrose was again not different from saline injected rats (data not shown).

**Conclusion**: Inhibiting MC receptors in the brain increases fat intake, whereas sugar intake is not affected.
