# Supplementary material for: Central Melanocortins Regulate the Motivation for Sucrose Reward
Source: PLoS One. 2015 Mar 26;10(3):e0121768. doi: 10.1371/journal.pone.0121768 (PMC4374756; doi:10.1371/journal.pone.0121768)
Supplement: S1 Table — (DOCX) [file pone.0121768.s003.docx]

**S1 Table:** Regions and accession numbers used for synthesis of the FISH probes

| Genes | Accession Number | Region |
| --- | --- | --- |
| DRD1 | NM_012546 | 29-2164 |
| DRD2 | NM_012547 | 410-1488 |
| MC3R | NM_001025270 | 198-1076 |
| MC4R | NM_013099 | 231-1136 |
